# One-pot resource-efficient synthesis of SnSb powders for composite anodes in sodium-ion batteries†‡

**DOI:** 10.1039/d0ra03679j

**Published:** 2020-06-10

**Authors:** Deming Tan, Peng Chen, Gang Wang, Guangbo Chen, Tobias Pietsch, Eike Brunner, Thomas Doert, Michael Ruck

**Affiliations:** Faculty of Chemistry and Food Chemistry, Technische Universität Dresden 01062 Dresden Germany michael.ruck@tu-dresden.de; Center for Advancing Electronics Dresden (CFAED), Technische Universität Dresden 01062 Dresden Germany; Max Planck Institute for Chemical Physics of Solids Nöthnitzer Str. 40 01187 Dresden Germany

## Abstract

SnSb alloy, which can be used as an anode in a sodium-ion cell, was synthesized following a resource-efficient route at low temperature. This one-pot approach greatly reduces the energy consumption and maximizes the efficient use of raw materials. The reaction of elemental tin and antimony in the ionic liquid (IL) trihexyltetradecylphosphonium chloride ([P_66614_]Cl) at 200 °C led to a microcrystalline powder of single-phase SnSb within 10 h with very high yield (95%). Liquid-state nuclear magnetic resonance spectroscopy revealed that the IL remains essentially stable during the reaction. It was recovered almost quantitatively by distilling off the organic solvent used for product separation. Composites of SnSb powder and carbon nanotubes (CNTs) were fabricated by a simple ball milling process. Electrochemical measurements demonstrate that the Na‖SnSb/CNTs cell retains close to 100% of its initial capacity after 50 cycles at a current of 50 mA g^−1^, which is much better than the Na‖SnSb cell. The greatly increased capacity retainability can be attributed to the conductive network formed by CNTs inside the SnSb/CNTs electrode, providing 3D effective and fast electronic pathways during sodium intercalation and de-intercalation.

## Abstract 2

SnSb powders were obtained by a highly efficient ionothermal process and successfully tested in a composite anode for sodium-ion batteries.

## Graphical abstract

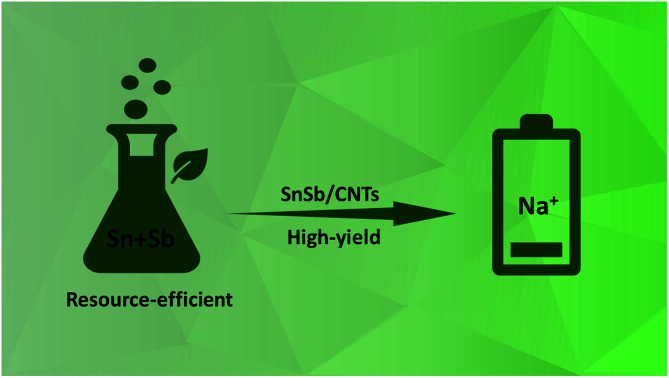

## Introduction

Secondary lithium ion batteries have been used in applications as variable as portable electronics, electric vehicles, and large-scale energy storage. Due to its technical and economic importance, a transition from non-renewable sources to cleaner and more sustainable energy storage methods is desirable.^1–4^ For the last few years the search for more abundant and lower cost energy storage has stimulated an influx of research into rechargeable sodium ion batteries.^5–11^ Based on the wide availability and low cost of sodium, sodium ion batteries offer the potential for meeting large scale grid energy storage needs. However, sodium ion batteries usually exhibit lower specific capacity, poorer cycling durability and rate capability due to the much larger ionic radius of Na^+^ (102 pm) as compared to Li^+^ (76 pm).^12,13^ The traditional graphite anode for lithium ion batteries does not perform well as a sodium ion battery anode. This leads to a demand for optimal Na-ion anodes with high capacity and long cycle life. Present investigations on sodium anode materials mostly focus on so called hard carbon, also known as non-graphitizable carbon, which can deliver a volumetric energy density of about 300 mA h g^−1^. However, hard carbon has certain drawbacks — notably, hard carbon exhibits poor cycling performance and the fact that the majority of its capacity is obtained at sodiation potentials close to metal plating, which is a safety hazard.

The recent surge of research on Na-ion anode materials provided strong motivation for the study of alloy anode materials because of their high volumetric and gravimetric capacities compared to hard carbon.^14–19^ Nevertheless, relatively low sodiation/de-sodiation potentials or large volume expansions oppose their commercial application. Among various potential metallic anode materials, Sn–Sb alloys have been studied extensively owing to their large theoretical capacities and inexpensiveness. The theoretical capacities for Na-ion storage in SnSb is 752 mA h g^−1^ based on the formation of Na_3.75_Sn and Na_3_Sb.^13^ An important challenge facing SnSb alloy anode is the severe volume change during cycling, resulting in pulverization of the active material, unstable solid-electrolyte interphase and peeling off of the active material from the current collector.^20^ These issues can greatly influence the electrochemical performance of the anodes and have considerably restricted their practical use. Therefore, it is of prime importance for maintaining the stable structure of the anodes upon sodiation/de-sodiation process. One idea was to design a matrix to absorb the mechanical stress induced by the huge volume changes and to form a conductive network inside the anode, such as carbon nanotubes (CNTs),^21^ carbon nanofibers,^22^ and carbon coating.^23^ These mentioned structures allow fast sodium ion transfer within the free void space around the active materials and accommodate the significant volume changes during cycling.

In previous works, the SnSb alloy has been synthesized with methods such as electrospinning, hot injection, chemical reduction, electrodeposition, and traditional solid-state means.^15,24–27^ These methods have their strengths, but most of them are fussy, which usually need considerable effort to conduct. Moreover, high yields have rarely been reported in these methods except for solid-state means, which, in turn, fail to provide nanoparticles. In order to maintain the momentum of future advances of Sn–Sb alloy chemistry, new simple resource-efficient synthetic routes are necessary. The recently developed ‘ionothermal’^28^ synthesis is regarded as a promising route.

The ionothermal synthesis, also called ionic liquids-assisted synthesis, makes use of the concept of conducting chemical procedures in an environmental-friendly way. Over recent years ionic liquids have received enormous interest in both the academic and the industrial fora.^29–31^ Most studies have focused on their ‘green’ chemistry attributes in conducting environmentally benign chemical procedures. Ionic liquids are salts in the liquid state, generally with melting point below 100 °C. The ions constituting ionic liquids have a low charge density and do not pack well together, resulting in low lattice energies and, consequently, low melting points. Compared with traditional solvents, ILs can deliver a lot of distinct advantages, such as negligible vapor pressures, good thermal stability as well as high synthetic flexibility, which make them suitable for applications in green and sustainable chemistry. Another major goal of conducting environmentally-friendly chemistry is to maximize the efficient use of raw materials and simultaneously to minimize waste.^32,33^ Therefore, the synthetic route has to address not only yield and purity of the final product but also the atom economy.

For the bimetallic Sn–Sb alloy system, the best way to conduct an atom economic synthetic route is to use elemental Sn and Sb as starting materials in order to minimize waste. However, when using the elements as starting materials, the conventional solid-state routes usually needs high temperature or prolonged annealing times due to low diffusion rates. The lowest reaction temperature of solid-state routes reported in literature is slightly above the melting point of Sn (m.p. 232 °C), at 240 °C or higher.^34,35^ However, annealing periods of more than one week or even several months are needed when applying this temperature range. Therefore, more commonly used reaction temperature is above the melting point of Sb (m.p. 631 °C), around 700 °C or higher.^13,25^ Generally solid-state routes are time consuming and costly. Here we report a simple ionothermal route to synthesize SnSb alloy directly from the elements at low temperature, which should also be up-scalable for industrial processes. This method combines moderate synthesis temperatures, a relatively short reaction time and a pressure-free setup of ionothermal synthesis^30,36,37^ with high atomic efficiency known from solid-state reactions. SnSb/CNTs composite electrodes are fabricated by mixing the obtained SnSb alloy with CNTs through a simple ball milling process. The electrochemical performance of a Na‖SnSb/CNTs half battery is also presented.

## Experimental

### Preparation of SnSb and SnSb/CNTs composite

#### Preparation of SnSb

All compounds were handled in an argon-filled glovebox (M. Braun; *p*(O_2_)/*p*^0^ < 1 ppm, *p*(H_2_O)/*p*^0^ < 1 ppm). Sb (Alfa Aesar, 99.9999%) were grind into fine powder before use. In a typical synthesis, 61.7 mg (0.52 mmol) of Sn (Fluka, 99.99%) and 59.66 mg (0.49 mmol) of Sb (Alfa Aesar, 99.9999%) were carefully weighed directly into a glass flask with a magnetic stirring bar. The starting materials were covered with 700 mg (1.3 mmol) [P_66614_]Cl (IoLiTec, 95%). The flask was closed with a glass stopper and subsequently placed in an oil bath. The flask was quickly heated to 200 °C and then stirred vigorously. After stirring for 10 h, the flask was removed from the bath and allowed to cool to room temperature. The solid products were collected by centrifugation and washed with ethanol three times and finally with distilled water twice. Afterward, the obtained powder was dried under dynamic vacuum at room temperature overnight. Almost quantitative (95%) yields of products were obtained.

#### Preparation of SnSb/CNTs composite

SnSb and MWCNTs (O.D. × L 10 nm × 5 μm, Sigma Aldrich, 98%) were mixed at a weight ratio of 9 : 1 and then ball milled (900 rpm) for 15 min.

### Powder X-ray diffraction

Powder X-ray diffraction (PXRD) of SnSb was performed at 296(1) K on an X'Pert Pro MPD diffractometer (PANalytical) equipped with a curved Ge(111) monochromator using Cu-Kα_1_ radiation (*λ* = 154.056 pm).

### Nuclear magnetic resonance spectroscopy

Liquid-state NMR experiments were performed using a Bruker Avance 300 MHz spectrometer with a 10 mm high-resolution probe. Reacted [P_66614_]Cl samples were collected from the upper part of the reaction mixture after cooling to room temperature. The ^119^Sn NMR spectra were recorded at the resonance frequency of 111.92 MHz using 512 scans, a relaxation delay of 5 s and a pulse length of 10 μs. The chemical shifts are referenced to SnMe_2_ (*δ* = 0 ppm). For calibration, the secondary external standard SnCl_4_ (*δ* = −150 ppm) was used. The ^31^P spectra were recorded at the resonance frequency of 121.49 MHz using 128 scans, a relaxation delay of 10 s and a pulse length of 8.3 μs. The chemical shifts in the ^31^P spectra were referenced relative to H_3_PO_4_. The spectra of the reacted [P_66614_]Cl samples were measured at 320 K.

### SEM and energy-dispersive X-ray analysis

Samples of the as-prepared dry SnSb and SnSb/CNTs were sprinkled on carbon adhesive. The carbon adhesive was fixed on a sample holder. SEM images were recorded on a field emission scanning electron microscope (FESEM, Zeiss Gemini 500, *U*_e_ = 1 kV). The compositions of the SnSb were determined by quantitative EDX analysis using a Zeiss Gemini 500 instrument equipped with an Oxford EDX detector.

### Preparation of hybrid electrolyte, SnSb/CNTs and SnSb electrode

The hybrid electrolyte was prepared in an Ar-filled glove box with H_2_O and O_2_ content below 0.1 ppm. 1 m NaClO_4_ (Sigma Aldrich, 98%) was added into propylene carbonate to form a solution, and then fluoroethylene carbonate was added into the solution with 10% weight percentage. The mixture was stirred in the glove box for another 8 h, producing transparent liquid. The SnSb/CNTs electrode was prepared according to the following procedure: SnSb/CNTs was mixed with Super P and alginate Na binder in a weight ratio of 7 : 2 : 1 with water as solvent. The resultant slurry was then cast onto a stainless-steel foil (304, 11 ± 1 μm in thickness, current collector). The SnSb/CNTs electrodes were first dried at room temperature and then at 80 °C for 8 h under vacuum. The mass loading of sample was 1.792 mg (∼2.3 mg cm^−2^), and the thickness determined by SEM was 27 μm (Fig. S1, ESI‡). A chunk of metallic Na was cut and pressed into a disc for the cathode with a diameter of 10 mm and a thickness of ∼1 mm. Then, all sodium-ion batteries were assembled as CR2032 coin-type batteries in an Ar-filled glovebox with H_2_O and O_2_ content below 0.1 ppm. SnSb electrode preparation procedure was analogous to that of SnSb/CNTs electrode.

### Electrochemical measurements

The long cycle (current 0.05 A) and rate performance data of Na‖SnSb/CNTs half battery was collected on LAND CT2001A battery system within voltage window of 0.1–3 V. The CV measurements were performed on the CHI760E electrochemical SnSb/CNTs as the working electrode and metallic Na as the reference electrode in a potential window from 0.1 V to 3 V *vs.* Na^+^/Na with a sweep rate of 0.02 mV s^−1^.

### BET measurements

N_2_ adsorption–desorption experiments were conducted at 77 K on a Quantachrome SI-MP instrument.

## Results and discussion

**Fig. 1:**
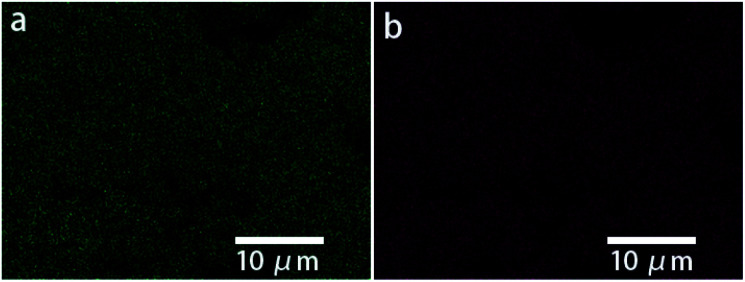
Elemental distribution of Sn (a) and Sb (b) according to their EDX signals.

**Fig. 2:**
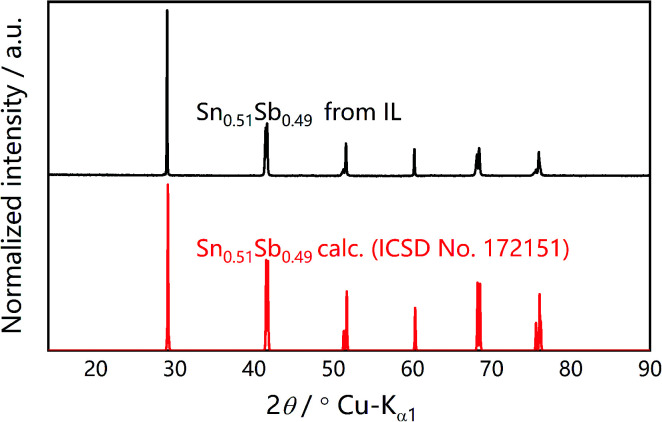
Measured PXRD pattern (Cu Kα_1_) of ionothermally synthesized SnSb (top) compared to the simulated pattern of Sn_0.51_Sb_0.49_ based on the Inorganic Crystal Structure Database (ISCD) entry no. 172151‡ (bottom).

**Fig. 3:**
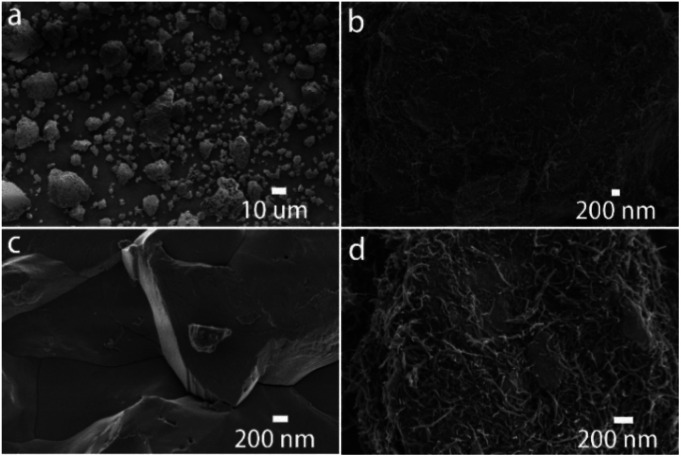
SEM image of the as-prepared SnSb (a and c) and of SnSb/CNTs composites (b and d).

In the novel ionothermal synthetic route to SnSb reported here, ionic liquid was used as a “green” solvent. These environmental-friendly solvents were often used in the preparation of crystalline materials in our previously reported works.^38–43^ In this study, the ionothermal route has greatly softened the synthesis conditions compared with conventional routes. Optimized reactions of elemental Sn and Sb in ionic liquid [P_66614_]Cl yield SnSb as the sole product. After a three-step processing by (i) ionic liquid removal with ethanol, (ii) thorough rinsing using distilled water, followed by (iii) drying the product, pure SnSb is obtained as a fine powder with a 95% yield. The synthesized SnSb powder were examined by powder X-ray diffraction (PXRD), energy-dispersive X-ray (EDX) spectroscopy and scanning electron microscopy (SEM). EDX spectra showed a homogenous distribution of Sn and Sb in the alloy (Fig. 1). The average weight percent of Sn and Sb was estimated to be 50.2 and 49.8 wt% (Fig. S2, ESI‡). Taking into account the accuracy of EDX elemental analysis, this corresponds to an equimolar, probably slightly Sn-rich composition. No P or Cl residuals of the IL were detected. The published phase diagrams of this binary system affirm a phase width for SnSb, but give different ranges. The PXRD pattern of our sample (Fig. 2) is in excellent accordance with literature data for Sn_0.51_Sb_0.49_ (rhombohedral, *R*3̄*m*, *a* = 4.325 Å, *c* = 5.338 Å)^21,35^ and shows no unindexed reflections, which also means that there are no detectable residuals of Sn, Sb or their oxides. SEM images of the SnSb powder show particles with sizes ranging from 1 to 30 μm and some aggregation (Fig. 3a and c).

SnSb/CNTs composites were obtained by ball milling the SnSb alloy with CNTs. The CNTs wrap the SnSb particles as can be seen in the SEM (Fig. 3b and d).

**Fig. 4:**
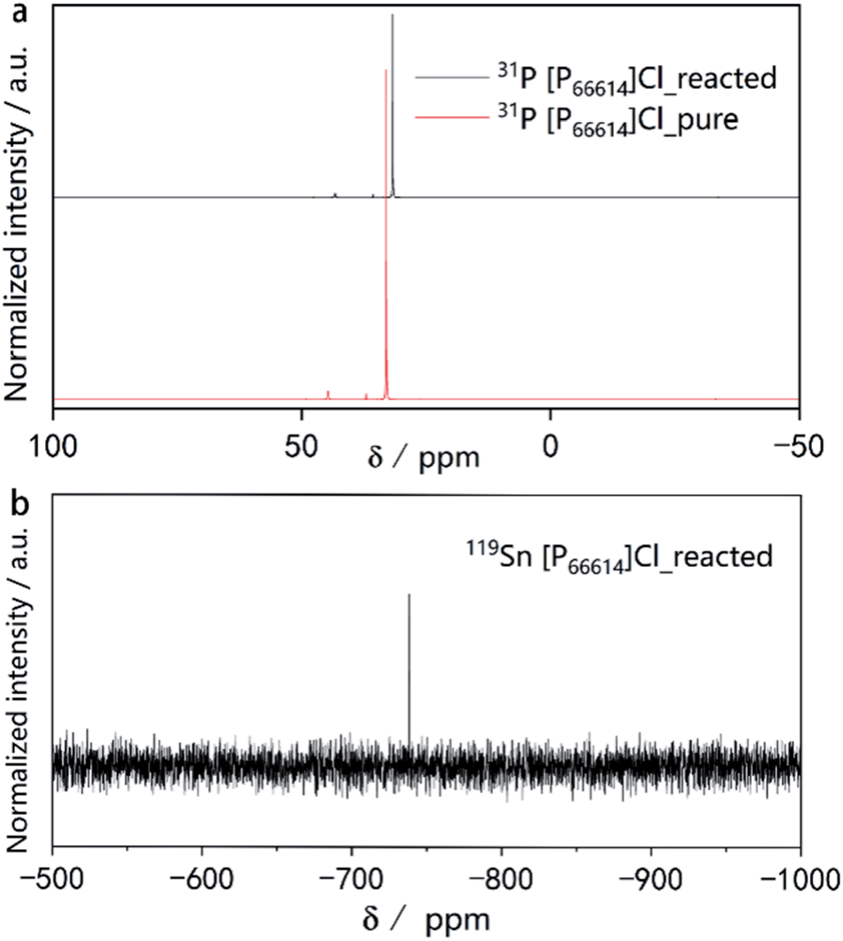
(a) ^31^P NMR spectra of pure [P_66614_]Cl before the reaction and after the reaction. (b) ^119^Sn NMR spectra of the [P_66614_]Cl solution after the reaction.

To obtain more insight into the ionothermal reaction, liquid-state ^31^P and ^119^Sn nuclear magnetic resonance (NMR) experiments were performed. A comparison of the ^31^P-NMR spectra of the IL before use as well as after the reaction (Fig. 4a) indicates no additional or missing signals, which means [P_66614_]Cl remained stable throughout the reaction. Moreover, no color changes of the ionic liquid were observed during the reaction. Due to the excellent stability of [P_66614_]Cl during the reaction, it can be recovered almost quantitatively by distilling off the organic solvent used for product separation. The simple setup, highly eco-friendly production processes, coupled with the high atom economic transformation, make this synthetic route promising for industrial processes.

The ^31^P spectra of [P_66614_]Cl after the reaction show slightly upfield shifted resonances. This provides a first hint for the mechanism of the reaction. As the reaction is quantitative according to the PXRD, but the powders of the starting materials do not dissolve visibly, there must be a transport step involving a dissolved intermediate species in the IL followed by a solid-state diffusion process resembling conventional solid-state reactions.^35,44^ Our previous work revealed that traces of HCl in [P_66614_]Cl cause a surface activation of elemental copper, promoting the formation of Cu_3−*x*_P.^45,46^ There is evidence for a similar activation process for the underlying reaction. However, the exact mechanism and the question of whether the IL contributes to the activation of the reactive species must be investigated in more detail in the future. The NMR signal at *δ*(^119^Sn) = −738 ppm in the ^119^Sn spectrum (Fig. 4b) indicates the presence of small amounts of Sn species in the reacted IL. As reported in literature, the signal found at −738 ppm can be associated with [SnCl_6_]^2−^.^47^ There are two explanations for the upfield shift in the ^31^P spectra.

(1) The shift might originate from the removal of Cl^−^ ions from ion aggregates in the IL, reducing the withdrawing of the electrons at the phosphors atom and increasing the shielding of the nuclei.

(2) Because the shift can is observed in the ^13^C and ^1^H NMR spectra and is consistent for all signals, it can also be assumed that [SnCl_6_]^2−^, Sn- or Sb-particles may change the magnetic susceptibility of the sample which in turn leads to the correspondingly shifted signals.^48,49^

We assume that HCl traces in the IL catalyze the oxidative dissolution of Sn to SnCl_6_^2−^. The transport species [SnCl_6_]^2−^ is reduced at the surface of an Sb particle forming highly reactive Sn atoms. The metal species that remain in the IL can explain the 5% yield deficit. The complementary reversible redox system, which must be present, is yet unknown.

**Fig. 5:**
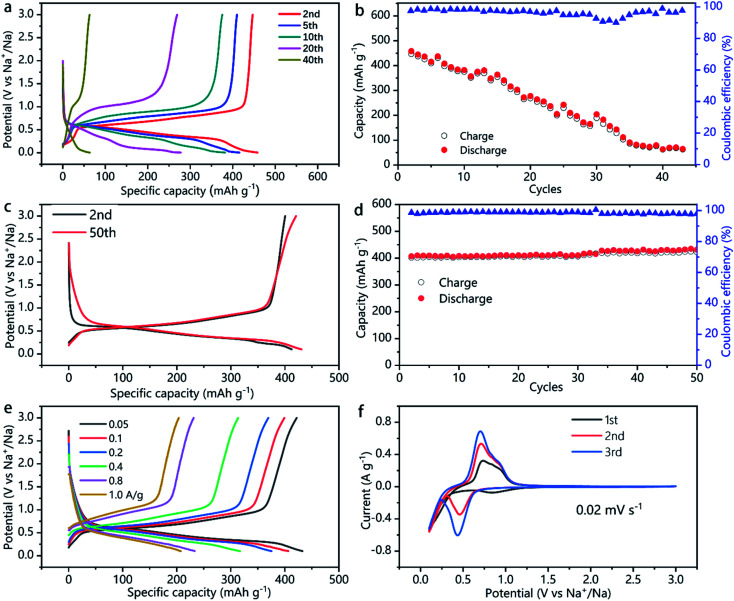
Electrochemical performance of SnSb and SnSb/CNTs electrode. (a) Rate performance of Na‖SnSb half battery. (b) Specific capacity and coulombic efficiency *versus* cycling number of SnSb electrode. (c) Long cycle performance of Na‖SnSb/CNTs half-cells at a current of 50 mA g^−1^. (d) Specific capacity and coulombic efficiency *versus* cycling number of SnSb/CNTs electrode. (e) Rate performance of Na‖SnSb/CNTs half-cell. (f) Cyclic voltammograms of SnSb/CNTs measured in Na-ion half-cells using a scan rate of 0.02 mV s^−1^ in the potential range of 0.1–3.0 V.

The capacity, energy density, rate capability, and the electrochemical process of the SnSb and SnSb/CNTs electrode studied in this work are shown in Fig. 5 and S3, S4, ESI.‡ Pure SnSb delivers an initial capacity of about 470 mA h g^−1^, but only 10% of its initial capacity retains after 40 cycles (Fig. 5a and b). This is probably because of the large volume changes during cycling, resulting in pulverization of SnSb. The powdery conductive agent Super P cannot form an effective conductive network to keep the active material SnSb in good conductivity during the volume changes, which lead to quick capacity decays.

In comparison, SnSb/CNTs, expected to combine the high capacity of SnSb with the good conductivity of CNTs, delivers an initial capacity of 422 mA h g^−1^. And the electrode can retain almost 100% of its initial capacity after 50 cycles, as is show in Fig. 5c and d. Table 1 presents the comparative electrochemical performance of SnSb synthesized here with SnSb reported in literature. As can be seen from the SEM image of SnSb/CNTs anode (Fig. 3b), the CNTs inside the electrode form a conductive network and SnSb is wrapped in it. The conductive network formed by CNTs can always maintain good contact with SnSb during the charge and discharge processes, providing 3D effective and fast electronic pathways during sodium intercalation and de-intercalation. Moreover, the powdery conductive agent Super P can fill the gap between the stripy CNTs and SnSb particles, to produce synergistic effects in promoting the conductivity of the electrode. These factors, which we discussed above, can explain the greatly increased capacity retainability of SnSb/CNTs electrode.

**Table 1:** Performance of selected SnSb Na-ion battery anodes

Materials	Current density (mA g^−1^)	Specific capacity (mA h g^−1^)	First cycle CE	Reference
SNSB	C/10	600	46%	15
SNSB NCS	200	560	<70%	13
RGO–SNSB	100	407	80.3%	50
SNS/SNSB@C	50	630	62%	20
SNSB	50	50–600	78.6%	This work
SNSB-10% CNT	50	422	75.5%	This work

In the first cycle, the high-surface-area conductive additives normally will consume a large amount of electrolyte associated with solid-electrolyte interface (SEI) formation.^13,19^ The first-cycle coulombic efficiency (CE) of SnSn–CNT was 75.5%, slightly lower than 78.6% of SnSb (Fig. S4, ESI‡). This is consistent with the result of a BET surface area determination (Fig. S5, ESI‡). Even though CNT addition increases the surface area to 27 m^2^ g^−1^, it does not deteriorate much the CE of electrodes. The second cycle CE of the SnSb/CNTs electrode is 97.2%, which even increased to 98–99.2% during subsequent cycles (Fig. 5d). During the subsequent cycles, a stable SEI is always kept and little side reactions and electrolyte decomposition occurs.

In addition to the good capacity retainability and high coulombic efficiency, the SnSb/CNTs electrode shows relatively low de-sodiation potentials (Fig. 5f), making it a promising candidate for full-cell applications. When charge current density ranges from 1.0 to 0.05 A g^−1^, SnSb/CNTs electrode exhibits specific capacity about 204–422 mA h g^−1^ (Fig. 5e).

Fig. 5f shows the cyclic voltammetry curves of the SnSb/CNTs electrode for the first three cycles at a scan rate of 0.02 mV s^−1^. The first scan is different from the following cathodic scan, which can be attributed to the formation of a SEI film on the surface of SnSb/CNTs electrode caused by some irreversible decomposition of the electrolytes. The SEI remained stable throughout subsequent cycles, and at the same time, the infiltration of the liquid electrolyte inside the SnSb/CNTs electrode also tends to stabilize. The reaction between SnSb/CNTs and Na are shown by the peaks at around 0.4 V (discharge, sodiation) and 0.6 V (charge, de-sodiation).^24^ As shown in Fig. 5c and e, the SnSb/CNTs electrode achieves most capacity contribution around 0.5–1 V.

## Conclusions

Phase-pure SnSb powders were synthesized by a resource-efficient environment-friendly approach. This synthesis pathway concerned not only with the aim of reducing the energy consumption and simplifying the reaction setup but also with the goal of maximizing the efficient use of raw materials and simultaneously minimizing waste. Typically, very high production yields of 95% were obtained. The ionic liquid was almost quantitatively recovered. SnSb/CNTs composites were fabricated by a simple ball milling process. The electrochemical performance of Na‖SnSb and Na‖SnSb/CNTs half-cell was also investigated. The Na‖SnSb/CNTs cell exhibits better electrochemical performance than Na‖SnSb cell in terms of long cycle and rate performance. The Na‖SnSb/CNTs cell can retain almost 100% of its initial capacity after 50 cycles at a current of 50 mA g^−1^. Considering the novel resource-efficient synthesis route and good electrochemical performance, SnSb/CNTs we report here is promising for practical applications in Na-ion batteries.

## Conflicts of interest

The authors declare no competing financial interest.

## Supplementary Material

RA-010-D0RA03679J-s001
