# Supplementary material for: One-pot resource-efficient synthesis of SnSb powders for composite anodes in sodium-ion batteries
Source: RSC Adv. 2020 Jun 10;10(37):22250–6. doi: 10.1039/d0ra03679j (PMC9054498; doi:10.1039/d0ra03679j)
Supplement: RA-010-D0RA03679J-s001 [file RA-010-D0RA03679J-s001.pdf]

Electronic Supplementary Material (ESI) for RSC Advances.
This journal is © The Royal Society of Chemistry 2020

One-pot resource-efficient synthesis of SnSb powders for composite anode in sodium-ion battery

Deming Tan,[a] Peng Chen,[a] Gang Wang,[a,b] Guangbo Chen,[a, b] Tobias Pietsch,[a] Eike Brunner,[a] Thomas Doert,[a] and Michael Ruck*[a,c]

[a]Faculty of Chemistry and Food Chemistry, Technische Universität Dresden, 01062 Dresden, Germany

[b]Center for Advancing Electronics Dresden (cfaed), Technische Universität Dresden, 01062 Dresden, Germany

[c]Max Planck Institute for Chemical Physics of Solids, Nöthnitzer Str. 40, 01187 Dresden, Germany

*Corresponding author: michael.ruck@tu-dresden.de

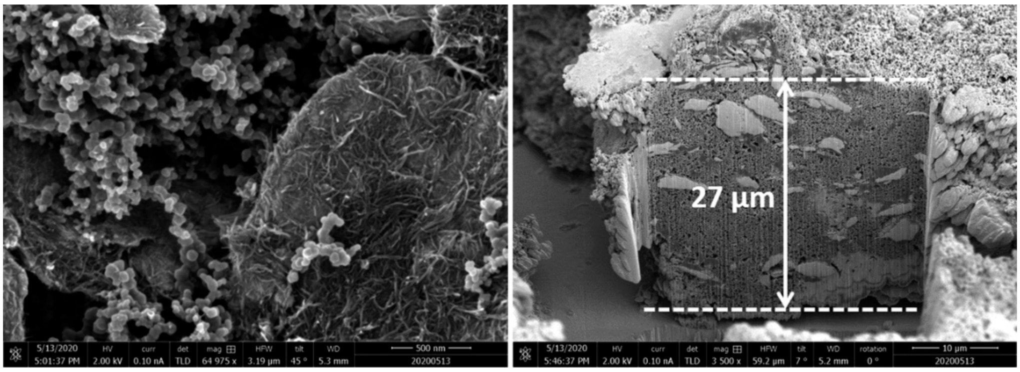

Fig.S1. The rock-like matter is SnSb, fiber matter is CNT. Particles are super P carbon additives. The average thickness of electrodes is 27 µm. The cross section was created by ion beam cutting.

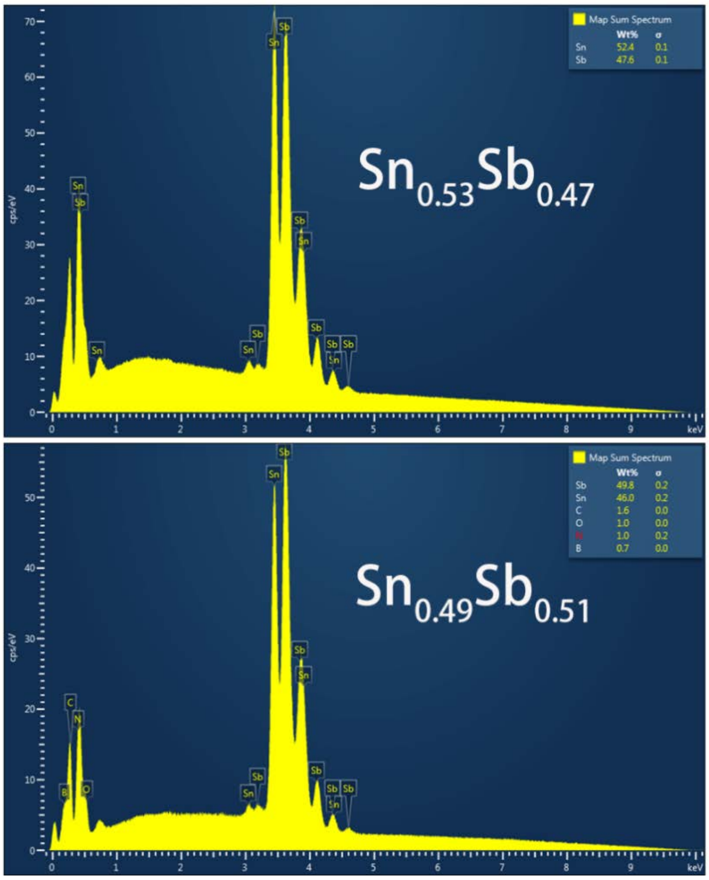

Fig. S2. EDX spectra of obtained SnSb. The average weight percent of Sn and Sb was estimated to be 50.2 and 49.8 wt.-%, which corresponds, with respect to the accuracy of EDX elemental analysis, to an equimolar, probably $Sn_{0.51}Sb_{0.49}$ composition.

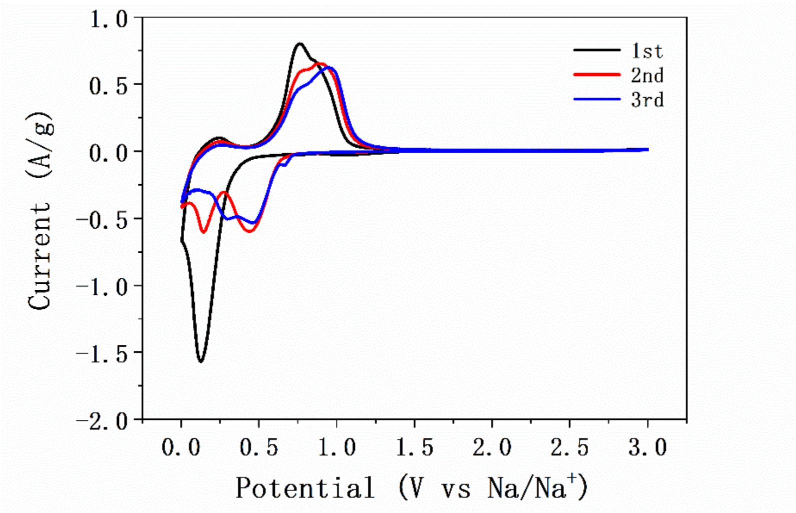

Fig. S3. Cyclic voltammograms of SnSb measured in Na-ion half-cells using a scan rate of 0.2 mV s^{-1} in the potential range of 0.005–3.0 V.

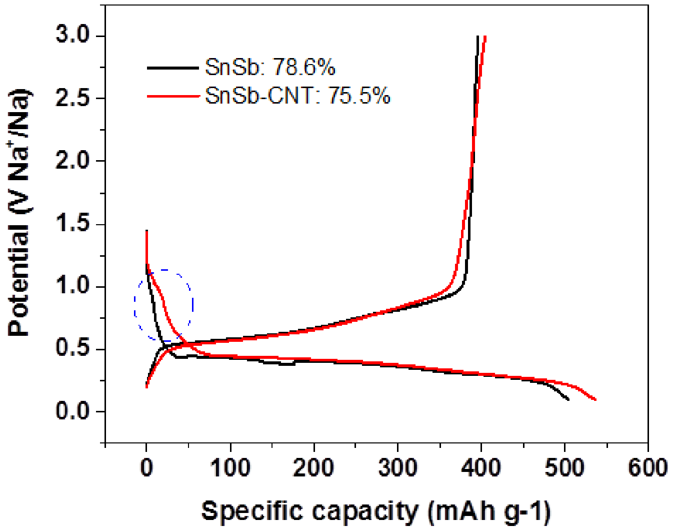

Fig. S4. The charge-discharge curves of SnSb and SnSb-CNT samples at the first cycle at 50 mA g^{-1}. The SnSb-CNT sample displays a little longer sloping discharge curve at 0.5-1.2 V than SnSb, indicating a little more electrolyte was consumed for SEI formation.

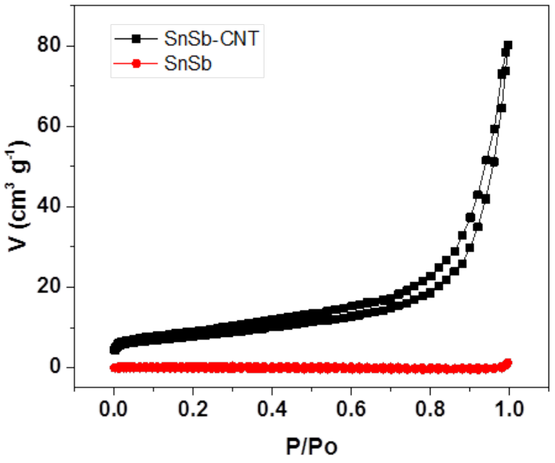

Figure S5. N_2 adsorption/desorption isotherms of SnSb and SnSb-CNT samples. SnSb-CNT delivers a BET surface area of 27 m^2/g, while the surface area of SnSb was too low to be reliably measured via a multipoint BET calculation method.
